# Phenotypic age acceleration as a novel predictor of benign prostatic hyperplasia: a prospective cohort study

**DOI:** 10.1007/s11357-025-01846-9

**Published:** 2025-09-11

**Authors:** Xuwen Li, Penghu Lian, Hongyan Chen, Liangzhe Zhang, Zhe Zhang, Jing Wang, Nianzeng Xing, Tao Jiang, Ziwei Chen, Xinlei Zhang, Xiongjun Ye

**Affiliations:** 1 Department of Urology, National Cancer Center/National Clinical Research Center for Cancer/Cancer Hospital Chinese Academy of Medical Sciences and Peking Union Medical College, Beijing, China; 2 Peking Union Medical College Hospital, Chinese Academy of Medical Sciences & Peking Union Medical College, Beijing, China; 3 Beijing ClouDNA Technology Co., Ltd., Beijing, China; 4 School of Electronic and Information Engineering, Beijing Jiaotong University, Beijing, China; 5 Medicine Innovation Research Division of Chinese PLA General Hospital, Beijing, China

**Keywords:** Benign prostatic hyperplasia, Phenotypic age, Phenotypic age acceleration, Biological aging, XGBoost

## Abstract

This study aims to investigate the predictive value of combined phenotypic age and phenotypic age acceleration (PhenoAgeAccel) for benign prostatic hyperplasia (BPH) and develop a machine learning-based risk prediction model to inform precision prevention and clinical management strategies. The study analyzed data from 784 male participants in the US National Health and Nutrition Examination Survey (NHANES, 2001–2008). Phenotypic age was derived from chronological age and nine serum biomarkers. PhenoAgeAccel, representing biological aging acceleration, was calculated as the residual from regressing phenotypic age on chronological age. Recursive Feature Elimination (RFE) identified 34 BPH-associated features, which were integrated into an XGBoost prediction model. Logistic regression evaluated PhenoAgeAccel-BPH associations, while SHapley Additive exPlanations (SHAP) quantified feature contributions to enhance model interpretability. The XGBoost model achieved an area under the curve (AUC) of 0.833 in the test set. Phenotypic age was strongly correlated with chronological age (*r* = 0.833), and individuals with PhenoAgeAccel exhibited a significantly elevated risk of BPH (*p* < 0.001). Adjusting the model with phenotypic age improved predictive performance (AUC = 0.853). SHAP analysis identified phenotypic age as the third most influential predictor (after trailing cancer history and lead exposure), highlighting its clinical relevance. Chronological age and serum biomarkers are critical predictors of BPH, while PhenoAgeAccel independently contributes to risk stratification. Integrating phenotypic age with machine learning provides a robust framework for the early detection of BPH and personalized risk assessment, aligning with advancements in aging biomarker research. This approach supports targeted interventions to mitigate BPH progression in aging populations.

**Supplementary Information:**

The online version contains supplementary material available at 10.1007/s11357-025-01846-9.

## Introduction

Benign prostatic hyperplasia (BPH) is a common disease of the urinary system affecting elderly men with a global incidence of approximately 94 million [1]. With the intensification of global aging, the absolute burden of BPH is rising at an alarming rate in most parts of the world [2, 3]. BPH has long been regarded as a benign disease; however, due to its long-lasting lower urinary tract symptoms, including frequent urination and urgency [4], the impairment of male erectile function [5], and its mediatory role in prostate cancer [6], BPH is gaining increasing levels of attention from clinicians and patients. Therefore, there is an urgent need to investigate the specific factors that contribute to the occurrence of BPH and predict its occurrence.

Age is considered a key risk factor for BPH [7]. Historically, age was simply regarded as the total time elapsed from an individual ‘s birth to the present day, in other words, chronological age. This explains the phenomenon that BPH predominantly affects elderly men. However, there is an increasing trend for BPH to affect younger individuals [2]; consequently, we cannot explain this condition simply by chronological age alone.

Diseases are manifestations of a certain systemic or local state during the aging process of organisms. Over recent years, some researchers have proposed the use of phenotypic age to reflect the aging state of organisms and replace chronological age in the study of diseases related to biological aging [8, 9]. Phenotypic age is usually calculated from chronological age and multiple clinical and biochemical biomarkers. Individuals whose phenotypic age is greater than their chronological age are considered to have positive phenotypic age acceleration (PhenoAgeAccel), while phenotypic age deceleration (PhenoAgeDecel) indicates that the individual ‘s phenotypic age is lower than their chronological age. Several studies have demonstrated that phenotypic age performs well in predicting the onset of diseases in multiple populations, and that PhenoAgeAccel represents an independent high-risk factor [8, 10].

Few studies have investigated the role of phenotypic age in the prediction of BPH, and there is a significant paucity of research focusing on the specific correlation between PhenoAgeAccel and BPH. In the present study, we investigated the role of phenotypic age in the prediction of BPH and defined the specific status of PhenoAgeAccel. In this study, we calculated phenotypic age based on chronological age and nine serum biomarkers associated with aging; then, we constructed a machine learning-based BPH prediction model. Our study found that phenotypic age can accurately predict the prevalence status of BPH in men. We hope that this study can contribute to the prevention and treatment of BPH.

## Materials and methods

### Study population

The data analyzed in this study were acquired from the National Health and Nutrition Examination Survey (NHANES) by the Centers for Disease Control and Prevention (CDC). We used NHANES data from 2001–2008 featuring detailed BPH information. BPH was identified based on self-reported questionnaire data. Specifically, participants who answered “Yes” to question KIQ141 (“Was it a benign enlargement?”) under the Prostate Conditions section were classified as having BPH. This question was a follow-up to an item asking whether the participant had ever been diagnosed with an enlarged prostate by a doctor or other health professional. For the 2003–2008 dataset, 784 participants were included after exclusion. The 2001–2002 dataset (228 samples lacking markers) was used as an external validation set for model performance.

### Assessment of phenotypic age and PhenoAgeAccel

Based on the phenotypic aging framework proposed by Levine et al. [11], phenotypic age was computed using the BioAge R package, which implements an algorithm incorporating ten clinically relevant variables: chronological age, albumin, alkaline phosphatase, creatinine, glucose, C-reactive protein, lymphocyte percentage, mean cell volume, red cell distribution width, and white blood cell count [12, 13].

PhenoAgeAccel was defined as a phenotypic age that was greater than the chronological age, while PhenoAgeDecel was defined as a phenotypic age that was lower than the chronological age.

### Dataset division

The dataset was divided into an 8:2 ratio; 80% of the data was used for model training and hyperparameter tuning, and the remaining 20% of the data was reserved as a holdout test set to evaluate the final model evaluation. Within the training set, fivefold cross-validation was employed to optimize feature selection and model development, thus ensuring robust and reliable performance.

### Feature selection

To identify the most relevant features associated with BPH, we performed Recursive Feature Elimination (RFE) in conjunction with Support Vector Machines (SVM) [14, 15]. This method recursively eliminates the least important features based on model performance, ultimately selecting the optimal subset of features for further analysis. The SVM classifier, which is known for its robust performance in high-dimensional spaces, was employed as the underlying model for feature ranking. The RFE algorithm iterates through all possible feature subsets, evaluating the performance of the model by cross-validation until the optimal feature set is identified.

### Development of machine learning for the prediction of BPH

To predict BPH, we evaluated the performance of three machine learning algorithms: Random Forest (RF), Logistic Regression (LR), and XGBoost (XGB). Following performance comparison, XGBoost was selected as the optimal model due to its superior accuracy and ability to capture intricate feature interactions. The model was then fine-tuned using grid search to optimize hyperparameters for improved performance. Scale_pos_weight was used to address class imbalance, and an early stopping strategy was employed to prevent overfitting. Other key hyperparameters included: n_estimators = 400, max_depth = 4, min_child_weight = 5, subsample = 0.2, colsample_bytree = 0.4, gamma = 0.2, learning_rate = 0.001, and eval_metric set to'logloss'.

### Model explainability

To assess the interpretability of the BPH prediction model, we employed SHapley Additive exPlanations (SHAP) values. SHAP values, based on cooperative game theory, can quantify the contribution of each feature to the predictive ability of a model, distributing the prediction among the features according to their marginal contributions [16]. This method enabled the identification of key features that influenced the outputs from our model. Positive SHAP values indicated features that increased the likelihood of BPH, while negative values highlighted features that reduced this likelihood.

### Statistical analysis

All statistical analyses were performed using R software (V4.1.1). The performance of the model was described by assessed accuracy (ACC), area under the curve (AUC), and a confusion matrix. Logistic regression was used to evaluate the association between PhenoAgeAccel and the risk of BPH, as well as to estimate the odds ratio (OR) and 90% confidence interval (CI).

## Results

### Participants

After excluding individuals with missing biomarker data, 784 participants were included in our final dataset for analysis. Of these, 621 were diagnosed with BPH. Table 1 describes the baseline characteristics of these participants. Mean chronological age was 69 ± 9.8 years in the PhenoAgeAccel group and 66 ± 10 years in the PhenoAgeDeceleration group. Notably, there was a 13-year difference in phenotypic age between the two groups. BPH was diagnosed in 78% of individuals in the PhenoAgeAccel group and 84% in the PhenoAgeDecel group.

**Table 1:** Baseline characteristics of participants with complete data (2003-2008 cohort)

	PhenoAgeAccel	PhenoAgeDecel	Overall
	(*N* = 616)	(*N* = 168)	(*N* = 784)
Chronological (years)			
Mean (SD)	69 (± 9.8)	66 (± 10)	68 (± 9.9)
PhenoAge (years)			
Mean (SD)	76 (± 12)	63 (± 10)	73 (± 13)
Sex			
Male	616 (100%)	168 (100%)	784 (100%)
Race			
Mexican American	62 (10%)	26 (15%)	88 (11%)
Non-Hispanic Black	118 (19%)	22 (13%)	140 (18%)
Non-Hispanic White	403 (65%)	108 (64%)	511 (65%)
Other Hispanic	22 (4%)	9 (5%)	31 (4%)
Other race—including multi-racial	11 (2%)	3 (2%)	14 (2%)
Education			
9–11th grade (includes 12th grade with no diploma)	80 (13%)	15 (9%)	95 (12%)
College graduate or above	142 (23%)	62 (37%)	204 (26%)
High school grad/GED or equivalent	136 (22%)	36 (21%)	172 (22%)
Less than 9th grade	90 (15%)	20 (12%)	110 (14%)
Some college or AA degree	168 (27%)	35 (21%)	203 (26%)
Marital status			
Divorced	55 (9%)	15 (9%)	70 (9%)
Living with partner	17 (3%)	7 (4%)	24 (3%)
Married	461 (75%)	120 (71%)	581 (74%)
Never married	23 (4%)	8 (5%)	31 (4%)
Separated	10 (2%)	2 (1%)	12 (2%)
Widowed	50 (8%)	16 (10%)	66 (8%)
Smoking			
Don’t know	1 (0%)	0 (0%)	1 (0%)
No	186 (30%)	64 (38%)	250 (32%)
Yes	429 (70%)	104 (62%)	533 (68%)
Alcohol frequency			
Mean (SD)	4.3 (± 20)	8.6 (± 42)	5.2 (± 27)
Missing	43 (7.0%)	8 (4.8%)	51 (6.5%)
BMI			
Mean (SD)	29 (± 5.6)	28 (± 4.4)	29 (± 5.5)
Missing	6 (1.0%)	0 (0%)	6 (0.8%)
C-reactive protein (mg/dL)			
Mean (SD)	0.50 (± 1.0)	0.094 (± 0.074)	0.41 (± 0.90)
White blood cell count (1000 cells/μL)			
Mean (SD)	7.2 (± 2.4)	5.7 (± 1.2)	6.9 (± 2.3)
Lymphocyte percent (%)			
Mean (SD)	27 (± 8.9)	31 (± 8.7)	28 (± 9.0)
Mean cell volume (fL)			
Mean (SD)	91 (± 5.0)	91 (± 4.4)	91 (± 4.9)
Red cell distribution width (%)			
Mean (SD)	13 (± 1.1)	12 (± 0.54)	13 (± 1.1)
Albumin (g/L)			
Mean (SD)	4.2 (± 0.32)	4.3 (± 0.29)	4.2 (± 0.32)
Serum glucose (mmol/L)			
Mean (SD)	6.1 (± 2.0)	5.3 (± 0.73)	5.9 (± 1.9)
Creatinine (μmol/L)			
Mean (SD)	100 (± 34)	84 (± 13)	97 (± 32)
Alkaline phosphatase (U/L)			
Mean (SD)	72 (± 32)	62 (± 16)	70 (± 30)
BHP status			
Non-BPH	136 (22%)	27 (16%)	163 (21%)
BPH	480 (78%)	141 (84%)	621 (79%)

### Screening of BPH-related features

**Fig. 1:**
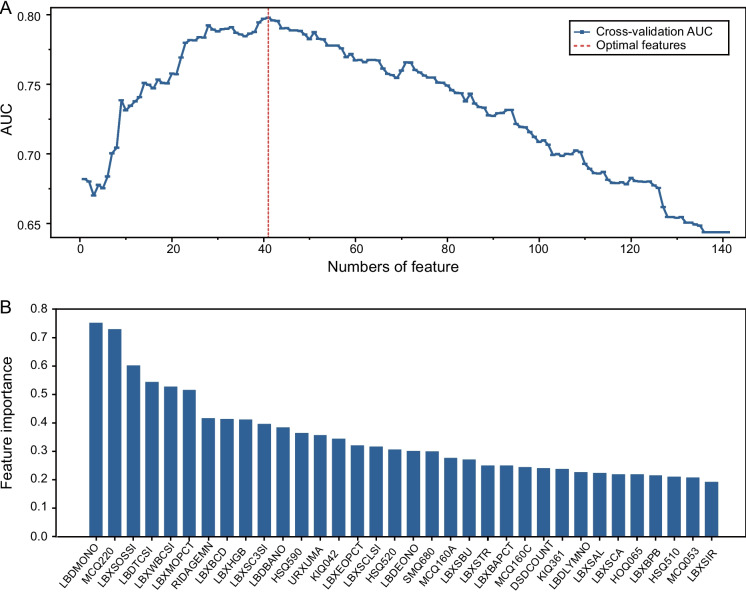
Recursive feature elimination (RFE) for BPH-related biomarker identification. **A** Iterative AUC optimization during RFE: The red vertical line marks the optimal feature count (*n* = 41) achieving peak discriminative performance. Features were ranked by permutation importance. **B** Feature importance bar plot: LBDMONO (Monocyte number), MCQ220 (Trailing cancer history), and LBXSOSSI (Osmolality) emerged as top predictors

AUC was employed as the evaluation metric to identify BPH-related features. Optimal model performance was achieved when the top 41 features were included (Fig. 1A). Following discussion with urology specialists, we excluded seven results that were irrelevant and finally retained 34 features that might be related to BPH (Table S1). Furthermore, we ranked these features according to their importance. As shown in Fig. 1B, the top five related features were LBDMONO, MCQ220, LBXSOSSI, LBDTCSI, and LBXWBCSI, which represent monocyte number, railing cancer history, osmolality, total cholesterol, and white blood cell count, respectively.

### Construction and validation of a predictive model for BPH

**Fig. 2:**
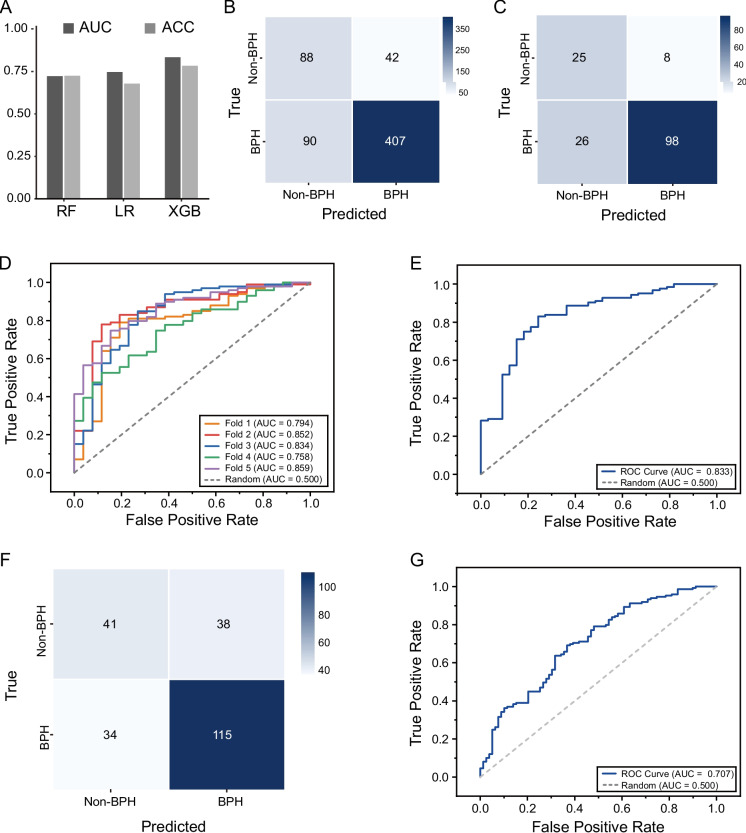
Machine learning model development and validation for BPH prediction. **A** Comparative performance of XGBoost vs. logistic regression/random forest models. XGBoost achieved superior test-set AUC (0.833 vs. 0.721–0.746) and ACC (0.783 vs. 0.678–0.724). **B–E** Model evaluation results for XGBoost, including confusion matrices (**B–C**) and ROC curves with AUC values (**D–E**) from fivefold cross-validation and test set. **F–G** External validation on an independent cohort (*n* = 228) confirmed robustness (AUC = 0.707), in line with internal validation results

Using the features identified earlier, we next constructed predictive models employing three commonly utilized machine learning techniques: Random Forest, Logistic Regression, and XGBoost. Compared with the other two methods, the XGBoost model outperformed in both AUC and ACC (Fig. 2A). Consequently, XGBoost was selected as the optimal method to construct the final predictive model. The XGBoost model was trained by fivefold cross-validation, and its performance was evaluated on the test set. Figure 2B shows a confusion matrix for the validation set, with a sensitivity of 0.819 and a specificity of 0.677. Figure 2D shows the results of fivefold cross-validation for the validation set, indicating that the AUC of the model ranged from 0.798 to 0.859, with an average AUC of 0.819. The model also demonstrated good performance in the test set data, with an AUC of 0.833 (Fig. 2C, E).

In the external validation cohort, the model exhibited moderate predictive performance. As illustrated by the confusion matrix (Fig. 2F), the model achieved a sensitivity of 0.772 and a specificity of 0.519. Although the AUC exhibited a slight reduction to 0.707 (Fig. 2G), the model maintained high sensitivity, thus highlighting its potential clinical utility for accurately identifying cases oh BPH.

### The relationship between PhenoAgeAccel and BPH

**Fig. 3:**
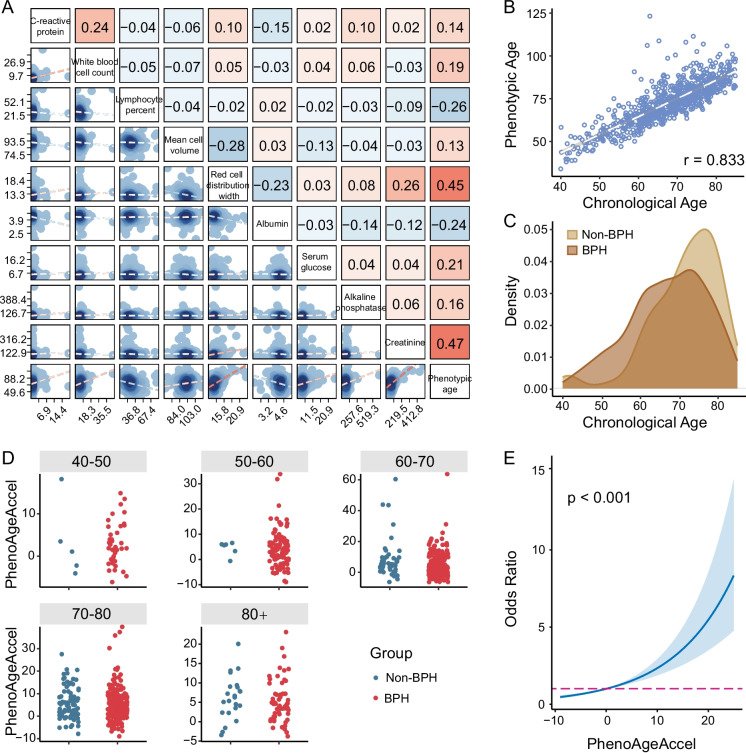
Phenotypic age dynamics and association with BPH risk. **A** Spearman correlation matrix: Phenotypic age showed moderate-to-strong correlations with serum creatinine (*r* = 0.47) and red cell distribution width (*r* = 0.45). **B** Linear regression (*r* = 0.833) between phenotypic and chronological age. **C** Density distribution of chronological age in BPH and non-BPH groups. The non-BPH group exhibits a right-shifted age distribution, with a higher proportion of older individuals. **D** BPH patients exhibited phenotypic age acceleration (mean Δ =  + 4.971 years, *p* = 0.013). **E** Logistic regression: PhenoAgeAccel increased BPH risk (OR = 1.08 per SD, 95% CI: 1.065–1.114)

Phenotypic age was calculated using nine blood biomarkers in conjunction with chronological age. The correlations between these biomarkers, as well as their individual relationships with biological age, are illustrated in Fig. 3A. Lymphocyte percent and albumin were negatively correlated with phenotypic age, with correlation coefficients of − 0.26 and − 0.24, respectively. In addition, the remaining biomarkers were all positively correlated. Among them, red blood cell distribution width and creatinine showed the strongest correlations (0.45 and 0.47, respectively).

In addition, we detected a strong positive correlation between phenotypic age and chronological age (*r* = 0.883). Next, we compared the distributional trends of chronological age and phenotypic age. Compared to the BPH group, the non-BPH group exhibited a greater density of participants in the advanced chronological age segments (Fig. 3C). To minimize the potential impact of the chronological age distribution, we then stratified the participants into several age groups according to their chronological ages: 40–50, 50–60, 60–70, 70–80, and 80 + years. For the 40–50, 50–60 and 60–70 age groups, the PhenoAgeAccel in the BPH group was higher than that in the non-BPH group, while in the 70–80 and 80 + age groups, there was almost no difference between the two groups (Fig. 3D).

Next, we constructed a logistic regression model to assess the relationship between PhenoAgeAccel and the probability of BPH. As PhenoAgeAccel increased, the OR progressively escalated, suggesting a positive correlation between higher PhenoAgeAccel and an increased likelihood of BPH (*p* < 0.001, Fig. 3E).

### Feasibility analysis of predicting BPH with phenotypic age

**Fig. 4:**
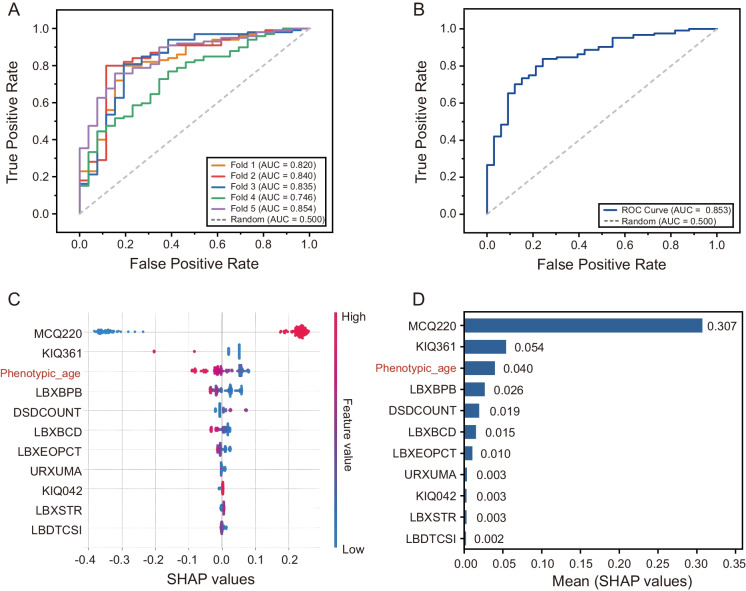
Interpretable risk stratification using phenotypic age-adjusted XGBoost model. **A–B** ROC curves demonstrating improved performance post-phenotypic age integration (ΔAUC =  + 0.020 in test-set). **C–D** SHAP summary plot: Phenotypic age (mean |SHAP|= 0.040) ranked third in global importance, trailing cancer history 0.307 and prostate check (0.054)

Building on these findings, we sought to explore whether combining phenotypic age could provide a more accurate prediction of BPH. We replaced chronological age with phenotypic age, while keeping all other features unchanged and applied the same strategy to train the XGBoost model. The mean AUC across fivefold cross-validation was 0.819 (Fig. 4A); the AUC for the independent test set was 0.853 (Fig. 4B), showing a slight improvement in predictive performance compared to the model based on chronological age.

To define the contribution of each variable in the prediction of BPH, we next applied SHAP on the XGBoost model to account for both the direct effect of a feature and its interactions with other features in the context of the model, thereby providing a more comprehensive and interpretable explanation of how each feature could influence the model’s predictions. A higher SHAP value indicated a greater contribution of the feature to predictive ability, signifying its more significant role in BPH prediction. Figure 4C, D shows the ranking of the top 11 variables according to their SHAP values. The results indicated that phenotypic age ranked the third in terms of importance in the model, while MCQ220 (Trailing cancer history) was identified as the main factor influencing the model. LBXBPB (Lead) was identified as the primary laboratory indicator.

## Discussion

BPH predominantly afflicts elderly men over the age of 60 years and has long been regarded as an age-related condition. Nevertheless, with the escalating global disease burden, it has become increasingly evident that a mere increase in age cannot account for the emerging trend of BPH affecting younger individuals [1, 2]. Aging represents a prominent research frontier, and an ever-growing number of diseases have been found to be intricately associated with the aging of the body and its tissues [9, 17–19]. On this foundation, certain researchers have put forward the utilization of phenotypic age to evaluate the aging process of the body and tissues, as well as their correlations with specific diseases. In this study, we systematically investigated the role of phenotypic age and its acceleration in the prediction of BPH. By integrating clinical biomarkers with machine learning methodologies, we identified the distinctive value of phenotypic age in the risk stratification of BPH.

In this study, we derived phenotypic age from the chronological age and nine serum biomarkers. We identified a profound connection between phenotypic age and BPH. Specifically, as PhenoAgeAccel advanced, the risk of developing BPH exhibited a progressive upwards trend. This finding aligns closely with the conclusions of two recently published studies. For example, Cheng et al. discovered that Han Chinese men with BPH had shorter telomere lengths in their white blood cells [20]. Another study revealed that epigenetic age contributed to the onset of lower urinary tract symptoms [21]. Moreover, the dose response relationship that we detected between PhenoAgeAccel and the risk of BPH bears a striking resemblance to previous research outcomes of diseases affecting in the cardiovascular and respiratory systems [8, 22]. This suggests that the acceleration of biological aging might serve as a common risk multiplier across various organ-related diseases.

Furthermore, we identified that among the population aged 40–70 years, the PhenoAgeAccel of BPH patients was higher than that of the non-BPH group. However, in the population aged over 70 years, the difference between the two groups was not pronounced (Fig. 3D). This phenomenon could potentially elucidate the epidemiological trend of the younger-onset of BPH. If younger individuals experience accelerated biological aging due to factors such as environmental stress and chronic inflammation, the aging process in their prostate may precede that corresponding to their chronological age. This serves as a reminder to clinicians that for young patients with micturition disorders, vigilance should be exercised to determine whether the case involves accelerated biological aging, rather than simply ascribing the condition to psychological or behavioral factors. For the elderly population (> 70 years), owing to the existence of the “aging ceiling effect” the marginal effect of biological aging diminishes. This phenomenon stems mainly from two aspects. Biologically, the accumulation of aging markers is characterized by a pattern that is initially rapid but then slows. A previous study by Marioni et al. [23] found that throughout the human life cycle, the accumulation of aging markers slows down significantly in the elderly. Therefore, after entering old age, the accumulation of aging markers enters a plateau (a ceiling value), which leads to reduced differences in biological aging status among individuals. Moreover, from a statistical perspective, most men over 70 years of age will have accumulated a large amount of aging-related damage, and the incidence of BPH increases significantly. Studies have shown that the incidence of BPH in the population over 70 years of age is approximately 80% [3], which to some extent weakens the impact of PhenoAgeAccel in the elderly population. Interestingly, a recent paper focusing on PhenoAgeAccel and the onset of dementia reported a decline in the efficacy of PhenoAgeAccel in the elderly population [19].

Traditional BPH research has predominantly centered on local indices such as prostate volume and PSA. In this study, we broadened this perspective to the overall systemic aging status by means of phenotypic age. Through correlation analysis, we determined that the red blood cell distribution width (RDW) and creatinine exhibited the most robust positive correlations with phenotypic age (*r* = 0.45, *r* = 0.47). This implies that systemic inflammation (as RDW reflects heterogeneity of the red blood cell production) and renal function impairment may be latent driving forces underlying BPH. Conversely, albumin was negatively correlated with the percentage of lymphocytes (Fig. 3A), thus supporting to the role of declining nutritional status and immunosenescence in prostate aging[24]. Notably, the monocyte count and the total white blood cell count ranked among the top five crucial factors for BPH (Fig. 1B), thus corroborating the classic hypothesis that chronic inflammation propels the development of BPH [25]. In fact, with the continuous advancement of research, it has become increasingly clear that the occurrence and development of BPH are not merely attributed to hyperplasia of the prostate parenchyma but rather represent a systemic alteration. In a previous study, researchers identified a close association between the concentrations of IL-2 and dimethyl lysine and BPH [26]. In addition, a recent prospective study discovered that high-density lipoprotein (HDL) cholesterol and apolipoprotein A are pivotal protective factors in preventing the progression of BPH [27]. These findings provide a theoretical foundation for predicting the occurrence and development of BPH through early-stage biological markers and for alleviating BPH via anti-inflammatory and nutritional support.

The BPH prediction model constructed by phenotypic age had an AUC of 0.853 in the test dataset, marginally surpassing the model that utilized chronological age. This outcome is congruent with the dominant hypothesis over recent years that phenotypic age outperforms chronological age when predicting the risk of disease [28]. SHAP analysis further indicated that phenotypic age ranked third in terms of model contribution (Fig. 4C), second only to the trailing cancer history (MCQ220) and lead exposure (LBXBPB). This suggests that phenotypic age not only exerts an independent influence on the risk of BPH but may also exacerbate disease progression by interacting with other factors, such as inflammation and metabolic disorders. In the traditional screening paradigm centered around chronological age, some young patients experiencing biological aging are prone to being overlooked. The incorporation of phenotypic age can optimize risk stratification, rendering it particularly suitable for the precise prevention and control of the trend of BPH affecting younger individuals.

The BPH indicator only appeared in the NHANES questionnaire from 2001 to 2008, and the non-BPH group had a higher mean chronological age. Therefore, the sample size and the age distribution of participants represent the main Limitations of this study. Although external validation was conducted using the temporally distinct 2001–2002 NHANES cycle, in contrast to the 2003–2008 data used for chronological age–based model development, the performance declined modestly (AUC reduced from 0.853 to 0.707). This attenuation may reflect underlying differences of sample characteristics (e.g., demographics, comorbidities, or data completeness), despite both datasets originating from the same national surveillance program. Nonetheless, our model exhibited stable discriminative capacity, with sensitivity remaining high in both the internal test set (0.798) and the external validation cohort (0.772). This emphasizes the need for future validation in fully independent external populations to confirm the robustness and generalizability of the model.

In summary, our findings demonstrate the potential of incorporating phenotypic age into risk stratification for BPH. Our model exhibited robust and consistent performance across internal and external datasets, despite modest performance attenuation likely attributable to cohort heterogeneity. These results support the utility of aging-related indicators in enhancing disease prediction beyond chronological age alone. Future studies incorporating multi-omics data are now warranted to further validate and refine the model, and to elucidate the underlying biological mechanisms driving BPH development and progression.

## Conclusions

PhenoAgeAccel is an independent and modifiable risk factor for BPH, with strong predictive value. It shifts clinical focus from chronological to phenotypic age metrics, enabling earlier risk assessment and personalized interventions. Biomarker-integrated models (e.g., albumin and CRP) can improve BPH prediction. Future multi-center studies should validate findings across populations and explore links between biological aging, inflammation, and hormonal factors to advance precision prevention strategies.

## Supplementary Information

Below is the link to the electronic supplementary material.

Supplementary file1 (XLSX 11 KB)

## Data Availability

The datasets used for the current study are available upon reasonable request from the corresponding author.

## Abbreviations

*PhenoAgeAccel*: Phenotypic age acceleration
*PhenoAgeDecel*: Phenotypic age deceleration
*BPH*: Benign prostatic hyperplasia
*NHANES*: National Health and Nutrition Examination Survey
*RFE*: Recursive feature elimination
*SVM*: Support vector machines
*SHAP*: SHapley Additive exPlanations
*RF*: Random forest
*LR*: Logistic regression
*XGB*: XGBoost
*ACC*: Assessed accuracy
*AUC*: Area under the curve
*OR*: Odds ratio
*CI*: Confidence interval
*RDW*: Red blood cell distribution width
*HDL*: High-density lipoprotein
